# Occurrence of Fungi and Fungal Toxins in Fish Feed during Storage

**DOI:** 10.3390/toxins12030171

**Published:** 2020-03-10

**Authors:** Constanze Pietsch, Georg Müller, Sulayman Mourabit, Simon Carnal, Kasun Bandara

**Affiliations:** 1 Institute of Natural Resource Sciences (IUNR), Zurich University of Applied Sciences, 8820 Wädenswil, Switzerland; georg.mueller@gmx.net (G.M.); sulayman.mourabit@zhaw.ch (S.M.); carnasim@students.zhaw.ch (S.C.); 2 Department of Fisheries & Aquaculture, Faculty of Fisheries and Marine Sciences & Technology, University of Ruhuna, 81000 Matara, Sri Lanka; kasun@fish.ruh.ac.lk

**Keywords:** aquaculture, feed contamination, toxin formation

## Abstract

Periods of unfavorable storing conditions can lead to changes in the quality of fish feeds, as well as the development of relevant mycotoxins. In the present study, a commercial fish feed was stored under defined conditions for four weeks. The main findings indicate that even storing fish feeds under unsuitable conditions for a short duration leads to a deterioration in quality. Mycotoxin and fungal contamination were subsequently analyzed. These investigations confirmed that different storage conditions can influence the presence of fungi and mycotoxins on fish feed. Notably, ochratoxin A (OTA) was found in samples after warm (25 °C) and humid (>60% relative humidity) treatment. This confirms the importance of this compound as a typical contaminant of fish feed and reveals how fast this mycotoxin can be formed in fish feed during storage.

## 1. Introduction

Correct and balanced nutrition is an essential requirement for fish welfare in aquaculture. However, even if the optimal nutritional composition of feeds is known, properly selecting feed ingredients is a major challenge. One factor often impairing feed quality is mycotoxin contamination [1,2], which poses a serious risk to animal farming [3]. Mycotoxin contamination is often related to the increased inclusion of plant material in feeds and differences in cereal processing [1]. Furthermore, humidity and temperature levels during the storage of feed ingredients and compounded feeds strongly influence the formation of mycotoxins. Furthermore, it is expected that climate change will intensify this problem in the future [4,5,6]. Besides potential impairment of the feed quality, e.g., due to decreasing vitamin levels and changes in the nutritional composition of the feeds [7,8], bioactive and toxic compounds including mycotoxins can be formed during feed storage [9,10]. The mycotoxins that are more likely to occur during food storage and in feed ingredients and compounded feeds often include aflatoxins and ochratoxins [11,12]. Ochratoxin A (OTA), which is often produced by *Aspergillus* and *Penicillium* species [13,14,15,16], is of major concern because it is known to have a detrimental impact on vertebrates, causing teratogenic, carcinogenic, and immune-modulative effects [3,17,18,19,20,21]. However, further fungal species are also known to occur in feed ingredients and animal feeds. These include the *Fusarium* and *Cladosporium* species, which produce common mycotoxins such as deoxynivalenol (DON) zearalenone (ZEN), and fumonisins, as well as less understood compounds, such as enniatins (ENNs), beauvericin, and the rarely described *Cladosporium*-derived toxins [5,22,23,24,25].

Currently, guidelines recommended by the EU as well as other countries with respect to mycotoxin contamination levels in animal feeds [26,27] cannot entirely protect fish from harm [28]. Moreover, it has not yet been determined whether short-term exposure to unfavorable storage conditions can lead to the increased production of mycotoxins in fish feeds. Therefore, the present study investigated how four weeks of storage under different conditions affected the microbial colonization of a commercial fish feed (which is described in detail in Section 5.1.) and the subsequent production of a set of relevant mycotoxins.

## 2. Results

### 2.1. Storage Conditions

Containers with a volume of 10 L each were selected for the experiments. The container humidity during the storage period was consistently kept higher than 61% relative humidity (rel H) in the ‘humid & cold’ treatment (samples 1–3) and higher than 62% rel H in the ‘humid & warm’ treatment (samples 7–9). The mean values are shown in Table 1. As a result, the final feed water content of these treatments ranged from 17.9% to 22.9%. In contrast, the humidity levels of the other two treatments were kept between 13.5%–21.7% and 12.0%–38.6% rel H (‘dry & cold’ treatment, samples 4–6, and ‘dry & warm’ treatment, samples 10–12, Table 1), which resulted in a feed water content that ranged from 6.4% to 8.8% following the treatments. The temperature data loggers confirmed that the mean temperatures for the ‘humid & cold’ and ‘dry & cold’ treatments were maintained at 8–9 °C, whereas in the ‘humid & warm’ and ‘dry & warm’ treatments, temperatures ranged at stable high values from approximately 25–27.5 °C.

### 2.2. Changes in the Nutritional Composition

**Figure 1:**
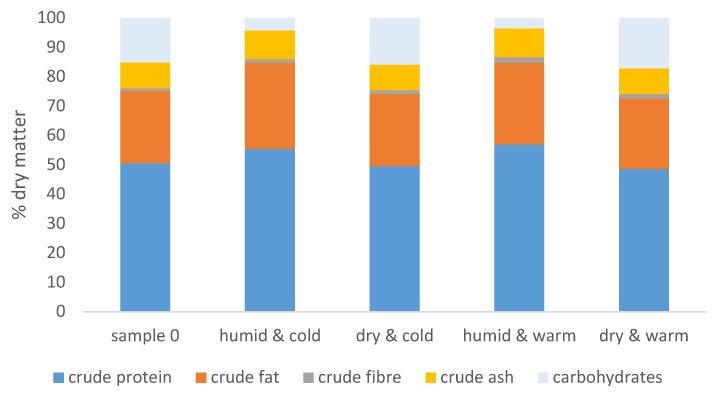
Nutritional composition of the experimental feed before and after storage for four weeks under different conditions described in Table 1, *n* = 3 for each storage condition.

After 30 days of storage, the feeds that had been stored under ‘humid & cold’ (samples 1–3) and ‘humid & warm’ (samples 7–9) conditions resulted in a decrease in the percentage of carbohydrates in the feed compared to the composition of the feed at start of the experiments (sample 0, Figure 1). 

The other two treatments (‘dry & cold’, samples 4–6, and ‘dry & warm’, samples 10–12) had no significant effect on the crude composition of the commercial feed.

### 2.3. Detection of Microorganisms on the Feeds

Different agarose plates yielded different fungal and bacterial colonies (Table 2). For most of the agarose plates that showed fungal growth, the number of colony forming units (cfu) was low (25–50 cfu/g feed).

**Figure 2:**
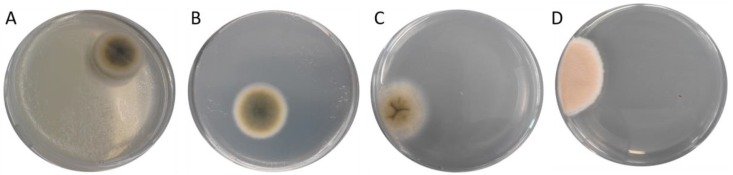
DG-18 agarose plates with different fungal colonies derived from different feed samples. Pictures taken after 96 h of plate incubation, **A** = sample 1, **B** = sample 6, **C** = sample 8, **D** = sample 9 as described in Table 1.

The cultivation of the different fish feed samples mixed with buffered peptone water on DG-18 plates, as described in detail in Section 5.3., favored the growth of colonies with a *Cladosporium*-like appearance that formed velvety, grey- or olivaceous-green colonies with a whitish edge (Figure 2A,B), as well as different light grey fungal colonies with a folded surface (Figure 2C) and orange-whitish cultures (Figure 2D).

**Figure 3:**
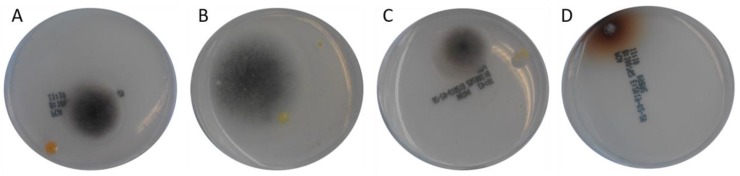
Fungal growth originating from inoculation of PCA agarose plates with different feed samples and subsequent examination after 96 h of incubation, **A** = sample 0, **B** = sample 4, **C** = sample 5, **D** = sample 12 as described in Table 1.

The cultivation of the different fish feed samples on PCA agarose plates resulted in the growth of fungi with a dark grey appearance (Figure 3). In addition, some yellow-colored bacterial colonies were observed on this plate type.

**Figure 4:**
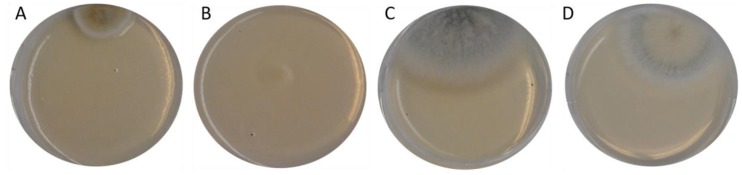
Fungal colonies grown on YGA agarose plates after 96 h of incubation of the plates inoculated with different feed samples, **A** = sample 5, **B** = sample 7 agarose replicate No. 1, **C** = sample 7 agarose replicate No. 2, **D** = sample 11 as described in Table 1.

The cultivation of the different fish feed samples on the YGA plates yielded a number of fungal colonies in different treatments with a *Fusarium*-like or *Cladosporium*-like appearance (Figure 4).

### 2.4. Fungal Identification via PCR

The analysis of genomic DNA was successful for almost all fungi that grew on the individual agarose plates and allowed for the identification of different fungal species in the fish feed samples (Table 3, Table 4, Table 5, Table 6 and Table 7).

However, the identification of the species based on beta tubulin sequences was not completely verifiable for some fungal samples. For this reason, the three blast hits with the highest identity of the isolated sequences are listed in Table 3, Table 4, Table 5, Table 6 and Table 7 with the NCBI library entries. The fungal material that was found in the feed before the experiment (sample 0) belonged to the genus *Cladosporium* and the closely related genus *Davidiella* (Table 3). 

The ‘humid & cold’ treatment (samples 1–3) resulted in the detection of similar *Cladosporium/Davidiella* species, as well as a fungus from feed sample 1 that showed the highest similarities to *Apioplagiostoma aceriferum*, a less often detected *Cladosporium* species (*Cladosporium acalyphae*), or an *Alternaria* species (Table 4). In addition, sample 3 showed growth of a fungi that was similar to the beta tubulin sequences of the *Sclerotinia* species. 

The ‘dry & cold’ treatment, samples 4–6, resulted in the growth of rather similar fungal colonies compared to feed samples 1–3, but also an *Aspergillus* species occurred on feed sample 4 (Table 5).

The ‘humid & warm’ treatment (samples 7–9) resulted in the detection of species in the fish feed samples belonging to the *Cladosporium/Davidiella* and/or *Aspergillus*, *Fusarium*, and *Penicillium* groups (Table 6). The fungus that grew on feed sample 6 on DG-18 plates looked like a *Cladosporium* species on the agarose plate, but the DNA content was too low for sequencing of the beta tubulin gene.

The ‘dry & warm’ treatment, samples 11–12, resulted in the detection of the *Cladosporium/Davidiella* species and an *Aspergillus* species in the cultured colonies on the respective agarose plates (Table 7). For sample 10, no fungal colonies could be observed on any of the agarose types.

### 2.5. Mycotoxin Analyses

After grinding, the feed samples were sent to an analytical lab and showed concentrations lower than the quantification limit for most of the assessed mycotoxins (Table 8). In addition, for DON, no relevant difference to the toxin concentration in the feed at the start of the experiment (sample 0) was observed. In contrast, the OTA content in the feed samples was increased in samples 7–9 due to the ‘humid & warm’ treatment in comparison to all the other samples.

The concentrations of ENNs in the feeds were analyzed at the start of the experiment and only in the ‘humid & warm’ samples (samples 7–9). Accordingly, a low amount of ENN B was detectable in sample 0 and a slightly higher value was measured in sample 8, but not in samples 7 and 9.

## 3. Discussion

### 3.1. Influence of Storage Conditions on the Nutritional Composition of the Feeds

Long-term storage is known to influence the chemical composition of animal feeds [8,29,30], but sufficient information on effects of shorter storage periods is lacking. Compared with the nutritional composition of the feed at the start of the experiments, storage for four weeks at humid conditions influenced the carbohydrate ratio in the fish feeds. This is typical for fungal growth on cereals, since fungi prefer to digest carbohydrates [31]. Other studies have also shown that fungal colonization influences the nutritional composition of fish feeds and, consequently, the growth performance of fish [32]. This is supported by Piotrowska et al. [33], who stated that *Penicillium*, *Mucor,* and *Eurotium* infestation in feed materials shorten the storage life of the feedstuff.

### 3.2. Microbiological Colonization of Feed Samples

Generally, the numbers for colony forming units (cfu) for the fungal colonies were low, and the fish feeds would have been suitable for animal feeding according to the VDLUFA guidelines [34]. The fungi that were observed on the agarose plates belonged to categories 4 and 5 of the guidelines, whereby especially the *Aspergillus* and *Penicillium* species belonging to category 5 are assumed to have an adverse effect on the quality of feeds. The number of growing microorganisms on the agarose plate type was slightly different for each treatment. Bacterial growth was observed on a number of agarose plates (namely on PCA plates for the ‘dry & cold’ treatment), but PCA plates are unselective plates and a number of different microorganisms can be cultured on them. In addition, the present study showed that mainly dark grey fungi belonging to the *Cladosporium/Davidiella* complex were obtained from the PCA plates, but their morphological appearance was different from what was observed on DG-18 plates. Therefore, the PCA seem to be unsuitable for broader fungal screening. The TSA-TCC plates yielded a number of fungal colonies, but these agarose plates are more suitable for the selective growth of bacterial colonies. The inclusion of chloramphenicol successfully suppressed bacterial growth on the YGA and Bengal Red agarose plates (Table 2), so these plate types appear to be the most suitable for detecting different fungal colonization on fish feeds.

### 3.3. Identification of Fungi

The typical fungi found in fish feeds are the *Aspergillus*, *Penicillium*, *Cladosporium,* and *Fusarium* species [35,36], which was confirmed by the present study. The occurrence of fungi in compounded animal feeds is certainly influenced by the selection of the ingredients and the milling process prior to feed production, since the *Alternaria*, *Cladosporium*, and *Fusarium* species have been reported to be more frequent on grain surfaces [23]. Furthermore, the presence of the *Aspergillus*, *Penicillium*, and *Cladosporium/Davidiella* species in the fish feeds confirmed previous reports [35,36], but the growth of the individual fungal species also varies with the storage conditions of the feed. The *Cladosporium/Davidiella* species that were identified in the feed at the start of the experiment were also identified in most of the subsequently treated feed samples. Moreover, the samples stored under cold conditions showed similarities in the occurrence of fungal colonies on the agarose plates, despite the detection of *Sclerotinia nivalis* in sample 3.

*Aspergillus fumigatus* was detected in 1 sample from the feed stored under colder conditions and in two samples from the treatments stored under warmer conditions. *A. fumigatus* favors warmer conditions for growth and mycotoxin production [37]. Thus, it was not surprising that a study from Egypt mostly identified the *Aspergillus* species, as well as *Penicillium bervicompactum, P. corylophylum, P. camemberti, P. quercetorum*, and *P. virdicatum,* and rather low aflatoxin levels in 25 feeds for Nile tilapia, *Oreochromis niloticus* [38]. Moreover, the less typical fungal species strain *P. chrysogenum* was identified in sample 9, as well as in in fish feed ingredients from East Africa [39]. Generally, *Aspergillus, Penicillium, Cladosporium,* and some *Davidiella* species can also occur as fungal contaminants in buildings [40], which may indicate that not all fungal spores that can be found in feed samples originate from the contamination of feed ingredients before harvest. Therefore, a more concise approach for an analytical study should include all feed ingredients and the resulting feed samples in order to describe the origin of the fungal contaminants in more detail.

### 3.4. Storage Conditions and Mycotoxin Formation

The optimal growth conditions of fungi differ for each species and, even more importantly, the optimal conditions for individual toxin production differ as well [33]. Therefore, the occurrence of fungal material does not necessarily mean that mycotoxin production also takes place [35]. 

Several mycotoxins may pose a risk to farmed fish [28], the most important of which was also included in the present study. The *Fusarium* species often occur on cereals [24] and show broad morphological differences and are therefore difficult to identify based on their appearance on agarose plates and their resistance to antimycotics [41]. The *Fusarium* species often prefer higher water activity (>0.86) and can grow at different temperatures depending on the species [42]. The *Fusarium merismoides* that were identified in sample 7 are a less common contaminant of food but have been found, for example, in potatoes [43], and have frequently been detected in soil samples [44,45]. In addition, this group appears to be close to the *Fusicolla* group, which has also been linked to the *Fusarium* species complex [46]. Surprisingly, the *Fusarium* species was rarely identified in the experimental feeds in the present study and typical *Fusarium*-related mycotoxins were therefore not detected at relevant concentrations in any of the feed samples. However, the capacity of the *Fusarium* species to produce mycotoxins may vary widely, and *Fusarium merismoides* may not have the biosynthetic genes to produce ZEN or trichothecenes [43,47].

In the present study, only the ‘dry & warm’ treatment resulted in significant formation of the OTA mycotoxin. High water activity and temperatures between 24–31 °C also favored OTA production in coffee [19]. However, the OTA concentrations that were reached in the experimental feeds from the ‘warm & humid’ treatment may be assumed as problematic for farmed fish [28]. Nevertheless, although it has been previously assumed that OTA commonly co-occurs with other mycotoxins in naturally contaminated feeds [28], this was not confirmed by the present study despite some minor occurrence of ENN B and DON. OTA commonly co-occurs with citrinin, but their production also depends on external factors such as light, despite the known regulation of temperature and water activity [48].

In temperate regions, OTA contamination is often linked to the occurrence of the *Penicillium verrucosum* infections, but it has been shown in warmer regions where the *Aspergillus* species, e.g., *A. carbonarius*, could also account for OTA production [3,18,19]. In addition, the ability of other *Penicillium* species to produce OTA has been reported in some studies or may have been based on possible incorrect identification of the *P. cyclopium, P. viridicatum,* and *P. chrysogenum* species [21,49]. However, *P. chrysogenum* was identified in sample 9 along with a small increase in OTA contamination after four weeks of treatment, which may indicate that some *P. chrysogenum* strains are capable of OTA formation. The ability of *P. chrysogenum* to produce OTA is supported by previous work from Zhang et al. [50].

The ingredients that are selected for fish feed production clearly influence the potential mycotoxin contamination of compounded feeds [28,51], and in addition to the results from the present study, it would have been interesting to use different fish feeds containing more plant-based ingredients for a similar study. The feed ingredients that often contain OTA are cereals or soybeans and corn [28]. However, these ingredients were not used at high percentages in the feed used for the present study. Therefore, the OTA concentration in the feed at the start of the experiment was low. Higher OTA concentrations were observed in the feed samples of the ‘warm & humid’ treatment after four weeks, and the formation of OTA in samples 7 to 9 could be related to the detection of the *Aspergillus* and *Penicillium* species, which are generally known to be capable of OTA production [18,19,20,21]. However, the ability of the *Aspergillus* species to produce mycotoxins has been related to the activity of certain genes [52], and *Aspergillus fumigatus* is known to produce mycotoxins such as gliotoxin and fumagillin [53] with a currently unknown impact on fish. 

It is also notable that the toxin concentrations in the same treatment may show considerable differences between the three replicates of the ‘warm & humid’ treatment. The heterogeneous distribution of OTA has already been described in stored grain. The reasons for these differences have been related to the fungal growth that commonly occurs at distinct hot spots. These provide conditions such as high water activity that supports fungal growth and toxin production. This variation causes considerable difficulty in measuring accurate OTA contamination levels in feed ingredients and feeds at the industrial level [18].

Mycotoxins produced by *Cladosporium* or the closely related the *Davidiella* species have rarely been described [5,54] and their effects on fish are unknown. Moreover, the fact that the two fungal colonies from samples 1 and 4 grown on DG-18 agarose plates were grouped as a *Cladosporium* or an *Alternaria* species is not entirely surprising, since according to more recent studies, at least some fungi previously sorted into the heterogeneous *Cladosporium* group should have been identified as the genus *Alternaria* instead [55]. However, the aforementioned fungal colonies from samples 1 and 4 may also belong to the genus *Apioplagiostoma aceriferum,* a fungal species that occurs on certain tree species [56,57]. *Alternaria* has been known to colonize on fish feeds [58], but similar reports for *Apioplagiostoma* are lacking. Moreover, the *Sclerotinia* species have been found on soybean, beans, canola [59], and linseed [60], which may indicate that the occurrence of this group of fungi in animal feed originates from contaminated feed ingredients. Seeing that this fungal group was only identified in sample 3 from the ‘cold & humid’ treatment, it could be related to the fact that *S. sclerotiorum* prefers temperatures lower than 20 °C [61].

Taken together, the findings in the present study show that the selection of feed ingredients and the storage conditions of the final feeds have a profound influence on the presence of fungal species on fish feeds. Nevertheless, a wider variety of fish feeds and mycotoxins should be investigated under different storage conditions.

### 3.5. Potential Effects on Fish

In vertebrates, including fish, OTA mainly affects the kidney and the liver, and OTA was also found to be teratogenic, carcinogenic, and immunotoxic [3,15,16,17,18,62]. Besides, embryotoxicity has been observed in amphibians and rodents, chicken, and fish [13,16,63,64,65,66]. Exposure by injection also had lethal consequences for rainbow trout with a concentration of 4.7 mg OTA per kg body weight being lethal to 50% of the animals [67]. Although not often conducted until now, some feeding experiments with different fish species have also shown detrimental effects of feed-borne OTA. Accordingly, channel catfish (*Ictalurus punctatus*), Nile tilapia, and sea bass (*Dicentrarchus labrax*) have shown reduced growth performance and survival at concentrations ranging from 300 to 4800 μg OTA per kg feed [62,68,69], whereas Atlantic salmon (*Salmo salar*) showed fast OTA elimination [70] and low sensitivity to this toxin [71]. In addition, Nile tilapia has also shown a change in the nutrient composition of the carcass [69].

The fungal species belonging to the *Cladosporium* group have not been described sufficiently, as this group is rather heterogeneous and polyphyletic, and contains several hundred different species at the moment [72]. *Cladosporium* are widely distributed in our environment [73]. It has been proposed that *Cladosporium* species prefer temperatures of less than 25 °C and humid culture conditions for growth and spore formation [74], which probably also applies to mycotoxin production. For the teleomorph *Cladosporium* species, the separate group *Davidiella* has been established [55]. Although no toxin production has been reported for the *Davidiella* species so far, the *Cladosporium* species are capable of producing a high variety of mycotoxins with mostly unknown consequences for farm animals [5]. The common occurrence of the *Cladosporium* species on the fish feed samples may, therefore, have more toxic consequences for fish than can be assumed by the confirmed occurrence of DON, ENN B, and OTA.

## 4. Conclusions

Fish feed storage under warm and humid conditions changes the quality of the feed and leads to the formation of mycotoxins. In the present study, the storage of a fishmeal-based fish feed under these conditions for one month resulted in a relevant amount of the mycotoxin OTA. Since OTA is a highly toxic mycotoxin, and based on current knowledge, which shows that it can even cause toxic effects in fish at an OTA concentration of approximately 20 μg/kg [28], this feed has to be assumed to be unsuitable for feeding to farmed fish. In a future study, a more plant-based fish feed should be selected for similar investigations, since aquaculture feeds increasingly contain plant materials.

## 5. Methods

### 5.1. Storage Conditions

For each treatment, 3.5 kg of a commercial trout feed (containing fishmeal, fish oil, wheat, wheat gluten, chicken offal meal, dextrose, wheat germ oil, linseed oil, blood meal, yeast, vitamins, and minerals, resulting in a composition as published by the manufacturer of 45% crude protein, 23% crude fat, and 8.8% water) were added to a 10-L plastic container. Four different storage conditions were chosen, which are shown in Table 9. The temperature in the treatment was maintained by adjusting the ambient temperature in the storage room. The humidity was adjusted by adding 350 g of water or using 500 g of silica gel per container. To prevent water loss from the containers, these were sealed with silicon. The relative humidity as% rel H and temperature values were monitored every 2 h using data loggers (iButton^®^, Maxim Integrated, San Jose, CA, USA). The values were monitored over the entire duration of the experiment, except for sample 10, which was monitored for only 22 days due to a subsequent loss of electricity that prevented further monitoring.

### 5.2. Nutritional Composition of the Feeds

The nutritional composition of the experimental diets was determined after drying the feed samples at 60 °C for 48 h followed by grinding. The lipid content of the fish feeds was determined using the Soxhlet method. Furthermore, the fish feeds were analyzed for dry matter (DM) (105 °C, until constant weight), and crude ash (550 °C, 12 h). The crude protein (according to the method VDLUFA 4.1.1. [75]) and crude fiber contents were determined by an external lab (Biolytix AG, Witterswil, CH). The carbohydrate fraction could then be estimated by subtraction of the nutrients mentioned above.

### 5.3. Detection of Microorganisms on the Feeds

Different agarose plates were used for cultivation of the microorganisms, since the agarose selection can also influence the growth of the bacterial and fungal species that can be achieved. Accordingly, Roti^®^-Aquatest plate PCA were used directly, whereas yeast-glucose-agarose (YGA) containing chloramphenicol, tryptone-soya-agarose (TSA) supplemented with 2,3,5-triphenyl-tetrazolium chloride (TTC), dichlorane-glycerine-agarose (DG-18), and Bengal Red agarose (containing chloramphenicol) plates were prepared in sterile petri dishes (material: PS, diameter: 5 cm) according to the supplier’s protocols (Carl Roth AG, Arlesheim, Switzerland). After solidification, the feed samples were applied. For this, 5 g of ground feed material was dispersed in 50 mL of buffered 0.1% peptone water. The samples were mixed, incubated at room temperature for 30 min, and centrifuged. A volume of 200 μL of the supernatant was then dispersed on the different agarose plates in duplicate. The plates were incubated in a Multitron Pro incubator (Infors AG, Bottmingen, Switzerland) at 25 °C for a maximum 96 h. After this, microbial colonization, the colony appearance, its structure and color was recorded, and the plates were stores at −20 °C for further analysis.

### 5.4. Fungal Identification via PCR

**Figure 5:**
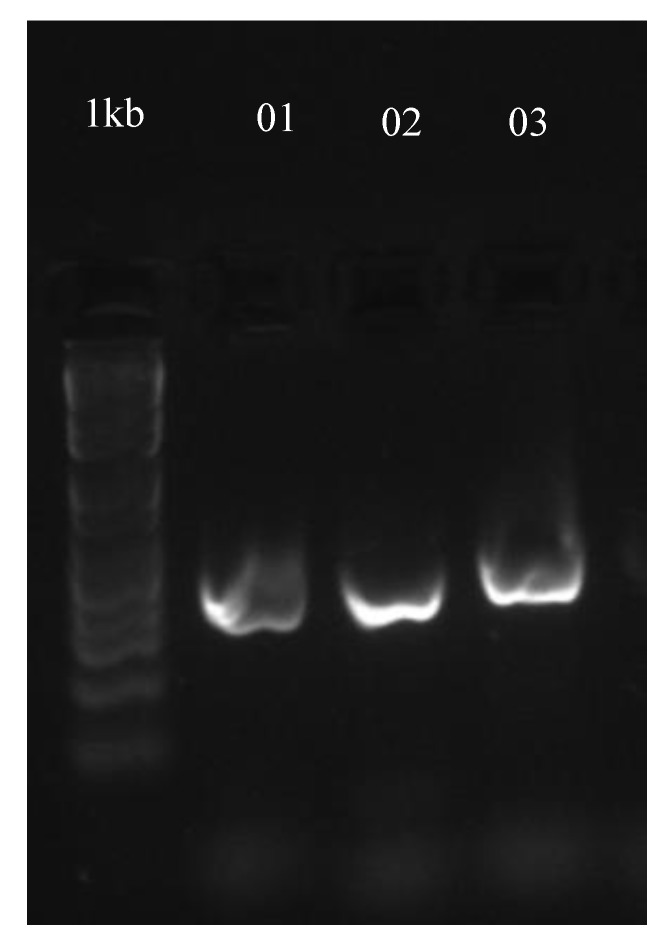
Agarose gel (1.5%) after gel electrophoresis at 80 V for 1 h containing the samples 1 and 4 in lane 01 and 02 analysed with the Bt1 primer set, and sample 9 in lane 03 analysed with the Bt2 primer set.

Genomic DNA was isolated using the DNeasy Plant Mini Kit (Qiagen AG, Hombrechtikon, Switzerland). The obtained DNA was used for PCR fungal species identification. For this, the kappa poymerase (Kappa Biosystems, Cape Town, South Africa) was used according to the manufacturers manual. As primers, the already published primer pairs Bt1 (5′-TTCCCCCGTCTCCACTTCTTCATG-3′ and 5′-GACGAGATCGTTCATGTTGAACTC-3′) and Bt2 (5′-GGTAACCAAATCGGTGCTGCTTTC-3′ and 5′-ACCCTCAGTGTAGTGACCCTTGGC-3′) [76] were included to analyze the beta tubulin region of ascomycetes. The PCR products were visualized on an 1.5% agarose gel containing GelRed^TM^ (Biotium, obtained from Chemie Brunschwig, Basel, Switzerland) after electrophoresis for 1 h at 80 V (Figure 5).

Prior to sequencing, the PCR products were purified using the NucleoSpin^®^ Gel and PCR Clean-up kits by Macherey-Nagel AG (Oensingen, Switzerland). The Sanger sequencing was performed by the Microsynth AG (Balgach, Switzerland) followed by blasting via the National Center for Biotechnology Information (NCBI, available at https://www.ncbi.nlm.nih.gov).

### 5.5. Toxin Analyses

At least 100 g of the ground feed samples were dried at 60 °C and sent to an analytical lab (Eurofins Scientific AG, Schönenwerd, Switzerland) for analysis of the different mycotoxins by means of a LC-MS/MS method. All toxin levels were calculated as μg per kg dry matter.

## Figures and Tables

**Table 1:** Summary of the mean temperature and humidity values (± SEM) maintained for each sample belonging to the different storage treatments for four weeks (except for the values of sample 10, which were monitored for only 22 days).

Treatment	Sample	Temperature (°C)	Humidity (% rel H)
	1	8.94 ± 0.03	76.2 ± 0.1
humid & cold	2	8.42 ± 0.02	79.1 ± 0.1
	3	8.39 ± 0.04	79.1 ± 0.1
	4	8.60 ± 0.02	18.4 ± 0.1
dry & cold	5	8.29 ± 0.04	26.5 ± 0.2
	6	8.55 ± 0.03	21.1 ± 0.2
	7	27.24 ± 0.20	70.1 ± 0.2
humid & warm	8	25.23 ± 0.14	73.9 ± 0.2
	9	26.34 ± 0.12	69.7 ± 0.1
	10	28.29 ± 0.17	30.3 ± 0.3
dry & warm	11	29.41 ± 0.22	20.5 ± 0.2
	12	25.75 ± 0.13	24.5 ± 0.2

**Table 2:** Summary of the fungal and bacterial colonization intensity of the different agarose plate types (± SEM) inoculated with the feed samples before and after storage under the conditions shown in Table 1. The agarose plates were maintained for 96 h at 25 °C before examination whereby the following scores were applied: -: no colony present, +: fungal or bacterial growth (i.e., usually 1–2 colonies on each plate) present on one of the replicate plates, ++: fungal or bacterial growth present on both of the replicate plates.

Sample	Bengal Red	DG18	PCA	TSA-TCC	YGA
	Fungi/Bacteria	Fungi/Bacteria	Fungi/Bacteria	Fungi/Bacteria	Fungi/Bacteria
0	-/-	-/-	+/+	+/-	-/-
1	+/-	+/-	-/-	-/+	+/-
2	+/+	-/-	-/+	-/+	-/-
3	-/+	+/-	-/+	-/-	-/-
4	+/-	+/-	+/++	-/+	-/-
5	-/-	-/-	+/++	-/-	+/-
6	-/-	-/-	+/-	+/-	-/-
7	++/-	-/-	-/+	-/+	++/-
8	-/-	-/-	-/+	-/-	-/-
9	+/-	++/-	-/+	-/+	-/-
10	-/-	-/-	-/+	-/+	-/-
11	-/-	-/-	+/-	-/-	-/-
12	-/-	-/-	+/+	+/-	-/-

**Table 3:** Summary of the identified fungal species from sample 0 taken at the start of the experiment and grown on the individual agarose types (Bengal Red, DG-18, PCA, TSA, YGA) and the sequence length and the results from the NCBI blast N of the fungi with the highest similarity (>95%) to the isolated sequence.

Sample	Agarose Type	Sequence Length	Related Fungal Species (NCBI Accession No.)
0	PCA	338 bp^2^	*Cladosporium* sp. strain CMW49962 (MH327787.1) *Davidiella tassiana* strain ATCC 66670 (EF101452.1) *Cladosporium allicinum* culture-collection CBS:177.71 (EF101451.1) (picture of the colony on the agarose plate shown in Figure 3A)
	TSA	348 bp^2^	*Davidiella tassiana* strain ATCC 66670 (EF101452.1) *Cladosporium* sp. strain CMW49962 (MH327787.1) *Cladosporium allicinum* culture-collection CBS:177.71 (EF101451.1)

^2^ PCR performed with the Bt2 primer set.

**Table 4:** Summary of the identified fungal species from samples 1 to 3 taken from the ‘humid & cold’ treatment and grown on the individual agarose types (Bengal Red, DG-18, PCA, TSA, YGA), and the sequence length and results from the NCBI blast N of the fungi with the highest similarity (>95%) to the isolated sequence.

Sample	Agarose Type	Sequence Length	Related Fungal Species (NCBI Accession No.)
1	DG-18	432 bp^1^	*Apioplagiostoma aceriferum* (AK110204.1) *Cladosporium acalyphae* strain SL-16 (KU574814.1) *Alternaria alternata* isolate CAU8331 (MH475293.1)
		350 bp^2^	*Cladosporium* sp. strain CMW49962 (MH327787.1) *Davidiella tassiana* strain ATCC 66670 (EF101452.1) *Cladosporium herbarum* strain BEOFB1812m (MH780976.1)
			(picture of the colony on the agarose plate shown in Figure 2A)
	YGA	358 bp^2^	*Davidiella tassiana* strain ATCC 66670 (EF101452.1) *Cladosporium* sp. strain CMW49962 (MH327787.1) *Cladosporium allicinum* culture-collection CBS:177.71 (EF101451.1)
2	Bengal Red	504 bp^2^	*Davidiella tassiana* strain ATCC 66670 (EF101452.1) *Cladosporium allicinum* culture-collection CBS:177.71 (EF101451.1) *Cladosporium* sp. strain CMW49962 (MH327787.1)
3	DG-18	395 bp^2^	*Sclerotinia nivalis* strain KGC-S0601 (JX296007.1) *Sclerotinia nivalis* isolate ZJ14 (KT023311.1) *Sclerotinia nivalis* isolate ZJ11 (KT023308.1)

^1^ PCR performed with the Bt1 primer set, ^2^ PCR performed with the Bt2 primer set.

**Table 5:** Summary of the identified fungal species from samples 4 to 6 taken from the ‘dry & cold’ treatment and grown on the individual agarose types (Bengal Red, DG-18, PCA, TSA, YGA), and the sequence length and results from the NCBI blast N of the fungi with the highest similarity (>95%) to the isolated sequence.

Sample	Agarose Type	Sequence Length	Related Fungal Species (NCBI Accession No.)
4	DG-18	425 bp^1^	*Apioplagiostoma aceriferum* (AK110204.1) *Cladosporium acalyphae* strain SL-16 (KU574814.1) *Alternaria alternata* isolate CAU8331 (MH475293.1)
		355 bp^2^	*Davidiella tassiana* strain ATCC 66670 (EF101452.1) *Cladosporium* sp. strain CMW49962 (MH327787.1) *Cladosporium allicinum* culture-collection CBS:177.71 (EF101451.1)
	PCA	139 bp^2^	*Aspergillus fumigatus* isolate 3 (MH536090.1) *Aspergillus fumigatus* strain m135 (MH208809.1) *Aspergillus fumigatus* strain m86 (MH208772.1) (picture of the colony on the agarose plate shown in Figure 3B)
		340 bp^2^	*Cladosporium* sp. strain CMW49962 (MH327787.1) *Davidiella tassiana* strain ATCC 66670 (EF101452.1) *Cladosporium allicinum* culture-collection CBS:177.71 (EF101451.1)
5	PCA	347 bp^2^	*Davidiella tassiana* strain ATCC 66670 (EF101452.1) *Cladosporium* sp. strain CMW49962 (MH327787.1) *Cladosporium allicinum* culture-collection CBS:177.71 (EF101451.1) (picture of the colony on the agarose plate shown in Figure 3C)
	YGA	367 bp^2^	*Davidiella tassiana* strain ATCC 66670 (EF101452.1) *Cladosporium allicinum* culture-collection CBS:177.71 (EF101451.1) *Cladosporium* sp. strain CMW49962 (MH327787.1) (picture of the colony on the agarose plate shown in Figure 4A)
6	DG-18		unknown (picture of the colony on the agarose plate shown in Figure 2B)
	TSA	341 bp^2^	*Cladosporium* sp. strain CMW49962 (MH327787.1) *Davidiella tassiana* strain ATCC 66670 (EF101452.1) *Cladosporium allicinum* culture-collection CBS:177.71 (EF101451.1)

^1^ PCR performed with the Bt1 primer set, ^2^ PCR performed with the Bt2 primer set.

**Table 6:** Summary of the identified fungal species from samples 7 to 9, taken from the ‘humid & warm’ treatment and grown on the individual agarose types (Bengal Red, DG-18, PCA, TSA, YGA), and the sequence length and results from the NCBI blast N of the fungi with the highest similarity (>95%) to the isolated sequence.

Sample	Agarose Type	Sequence Length	Related Fungal Species (NCBI Accession No.)
7	YGA	497 bp^2^	*Aspergillus fumigatus* isolate Z1201.1018 (KJ175518.1) *Aspergillus fumigatus* isolate M 169 (KJ175506.1) *Aspergillus fumigatus* (MF189897.1) (picture of the colony on the agarose plate shown in Figure 4C)
		353 bp^2^	*Davidiella tassiana* strain ATCC 66670 (EF101452.1) *Cladosporium* sp. strain CMW49962 (MH327787.1) *Cladosporium allicinum* culture-collection CBS:177.71 (EF101451.1) (picture of the colony on the agarose plate shown in Figure 4B)
	Bengal Red	360 bp^2^	*Cladosporium oxysporum* isolate 015 (MF175217.1) *Cladosporium oxysporum* isolate 056 (MF175222.1) *Cladosporium oxysporum* isolate 028 (MF175220.1)
		337 bp^2^	*Fusarium merismoides var. merismoides* strain F-266,788 (EU860027.1) *Fusarium merismoides* isolate N271A (KP710663.1) *Fusicolla septimanifiniscientiae* strain CBS 144935 (MK069408.1)
		471 bp^2^	*Davidiella tassiana* strain ATCC 66670 (EF101452.1) *Cladosporium allicinum* culture-collection CBS:177.71 (EF101451.1) *Cladosporium* sp. strain CMW49962 (MH327787.1)
		341 bp^2^	*Cladosporium* sp. strain CMW49962 (MH327787.1) *Davidiella tassiana* strain ATCC 66670 (EF101452.1) *Cladosporium allicinum* culture-collection CBS:177.71 (EF101451.1)
8	DG-18	333 bp^2^	*Cladosporium* sp. strain CMW49962 (MH327787.1) *Davidiella tassiana* strain ATCC 66670 (EF101452.1) *Cladosporium allicinum* culture-collection CBS:177.71(EF101451.1) (picture of the colony on the agarose plate shown in Figure 2C)
9	DG-18	401 bp^2^	*Penicillium chrysogenum* strain BEOFB11120m (MH780066.1) *Penicillium chrysogenum* isolate 186 (MH018233.1) *Penicillium chrysogenum* culture MUT<ITA>:2255 (MG832189.1) (picture of the colony on the agarose plate shown in Figure 2D)

^2^—PCR performed with the Bt2 primer set.

**Table 7:** Summary of the identified fungal species from samples 10 to 12 taken from the ‘dry & warm’ treatment and grown on the individual agarose types (Bengal Red, DG-18, PCA, TSA, YGA), and the sequence length and results from the NCBI blast N of the fungi with the highest similarity (>95%) to the isolated sequence.

Sample	Agarose Type	Sequence Length	Related Fungal Species (NCBI Accession No.)
10	-	-	-
11	YGA	481 bp^2^	*Aspergillus fumigatus* isolate Z1201.1018 (KJ175518.1) *Aspergillus fumigatus* isolate M 169 (KJ175506.1) *Aspergillus fumigatus* isolate 3 (MH536090.1) (picture of the colony on the agarose plate shown in Figure 4D)
12	PCA	501 bp^2^	*Davidiella tassiana* strain ATCC 66670 (EF101452.1) *Cladosporium allicinum* culture-collection CBS:177.71 (EF101451.1) *Cladosporium* sp. strain CMW49962 (MH327787.1) (picture of the colony on the agarose plate shown in Figure 3D)

^2^ PCR performed with the Bt2 primer set.—means that no fungal species could be detected.

**Table 8:** Summary of the produced mycotoxins (μg/kg) in the samples from the different treatments (as described in Table 1), DON = deoxynivalenol, ZEN = zearalenone, OTA = ochratoxin A, ENN = enniatin, FB = fumonisin, T2 = T2 toxin, HT2 = HT2 toxin. The LOQ values for each mycotoxin are included in brackets.

Mycotoxin (LOQ)	Aflatoxins ^1^ (2.5)	DON (12.5)	ZEN (12.5)	OTA (1.2)	ENN B ^2^ (12.7)	BEA (12.7)	FB_1_ + FB_2_ (50)	T2 + HT2 (25)
sample 0	<2.5	34.1	<12.5	<1.2	17.7	<13	<50	<25
sample 1	<2.5	39.8	<12.5	<1.2	n.d.	n.d.	<50	<25
sample 2	<2.5	27.0	<12.5	<1.2	n.d.	n.d.	<50	<25
sample 3	<2.5	37.0	<12.5	<1.2	n.d.	n.d.	<50	<25
sample 4	<2.5	34.9	<12.5	<1.2	n.d.	n.d.	<50	<25
sample 5	<2.5	28.4	<12.5	<1.2	n.d.	n.d.	<50	<25
sample 6	<2.5	33.8	<12.5	<1.2	n.d.	n.d.	<50	<25
sample 7	<2.5	30.0	<12.5	501.7	<12.7	<12.7	<50	<25
sample 8	<2.5	37.4	<12.5	7.1	18.5	<12.7	<50	<25
sample 9	<2.5	25.1	<12.5	5.7	<12.7	<12.7	<50	<25
sample 10	<2.5	33.4	<12.5	<1.2	n.d.	n.d.	<50	<25
sample 11	<2.5	43.0	<12.5	<1.2	n.d.	n.d.	<50	<25
sample 12	<2.5	28.1	<12.5	<1.2	n.d.	n.d.	<50	<25

^1^ including aflatoxin B_1_, B_2_, G_1_ and G_2_, ^2^ ENN A, ENN A_1_, and ENN B_1_ were also analyzed, but showed values <LOQ.

**Table 9:** Treatment of the samples.

Sample	Treatment	Duration of Exposure (Days)
0	none	none
1		32
2	humid & cold	32
3		32
4		32
5	dry & cold	32
6		32
7		30
8	humid & warm	30
9		30
10		30
11	dry & warm	30
12		30
